# Deletion of the glycosyltransferase *bgsB of Enterococcus faecalis *leads to a complete loss of glycolipids from the cell membrane and to impaired biofilm formation

**DOI:** 10.1186/1471-2180-11-67

**Published:** 2011-04-06

**Authors:** Christian Theilacker, Irina Sava, Patricia Sanchez-Carballo, Yinyin Bao, Andrea Kropec, Elisabeth Grohmann, Otto Holst, Johannes Huebner

**Affiliations:** 1 Center for Infectious Diseases and Travel Medicine, University Medical Center Freiburg, Germany; 2 Division of Structural Biochemistry, Research Center Borstel, Leibniz-Center for Medicine and Biosciences, Germany

## Abstract

**Background:**

Deletion of the glycosyltransferase *bgsA *in *Enterococcus faecalis *leads to loss of diglucosyldiacylglycerol from the cell membrane and accumulation of its precursor monoglucosyldiacylglycerol, associated with impaired biofilm formation and reduced virulence in vivo. Here we analyzed the function of a putative glucosyltransferase EF2890 designated *biofilm-associated glycolipid synthesis B (bgsB) *immediately downstream of *bgsA*.

**Results:**

A deletion mutant was constructed by targeted mutagenesis in *E. faecalis *strain 12030. Analysis of cell membrane extracts revealed a complete loss of glycolipids from the cell membrane. Cell walls of 12030Δ*bgsB *contained approximately fourfold more LTA, and ^1^H-nuclear magnetic resonance (NMR) spectroscopy suggested that the higher content of cellular LTA was due to increased length of the glycerol-phosphate polymer of LTA. 12030Δ*bgsB *was not altered in growth, cell morphology, or autolysis. However, attachment to Caco-2 cells was reduced to 50% of wild-type levels, and biofilm formation on polystyrene was highly impaired. Despite normal resistance to cationic antimicrobial peptides, complement and antibody-mediated opsonophagocytic killing in vitro, 12030Δ*bgsB *was cleared more rapidly from the bloodstream of mice than wild-type bacteria. Overall, the phenotype resembles the respective deletion mutant in the *bgsA *gene. Our findings suggest that loss of diglucosyldiacylglycerol or the altered structure of LTA in both mutants account for phenotypic changes observed.

**Conclusions:**

In summary, BgsB is a glucosyltransferase that synthesizes monoglucosyldiacylglycerol. Its inactivation profoundly affects cell membrane composition and has secondary effects on LTA biosynthesis. Both cell-membrane amphiphiles are critical for biofilm formation and virulence of *E. faecalis*.

## Background

The properties of the bacterial cell envelope are pivotal for the interaction of bacteria and the host organism [1]. *Enterococcus faecalis *expresses several cell-wall glycopolymers that make up the cell envelope, including capsular polysaccharides [2], cell-wall carbohydrates [3], cell-wall teichoic acid, lipoteichoic acid (LTA) [4], and glycolipids [5]. We have recently constructed a deletion mutant of the glycosyltransferase *bgsA *in *E. faecalis *[5]. Deletion led to a profound shift of the equilibrium of the two main cell wall glycolipids: monoglucosyldiacylglycerol (MGlcDAG) accumulated in the cell membrane of the *bgsA *mutant, while the production of diglucosyldiacylglycerol (DGlcDAG) was completely abrogated [5]. The *bgsA *mutant displayed normal cell morphology and growth characteristics but was impaired in attachment to colonic epithelial cells, and biofilm formation was almost completely abolished [5]. Remarkably, the LTA content of the mutant was higher due to the increased length of the glycerol-phosphate polymer.

The role of glycolipids in membrane physiology has been investigated in the cell wall-less bacterium *Acholeplasma laidlawii*, which produces glycolipids that are chemically identical to MGlcDAG and DGlcDAG of *E. faecalis *[6,7]. In *Acholeplasma*, the ratio of DGlcDAG to MGlcDAG governs the lipid bilayer's elasticity, curvature, and surface-charge density [6-8]. Interestingly, the pathway of glycolipid synthesis is highly conserved, and the type 4 family of NDP-glucose glycosyltransferases contains 107 UDP-sugar glycosyltransferases of bacterial, fungal, and plant origin [9]. Aside from their role as cell membrane components, glycolipids are also involved in the synthesis of LTA in bacteria with low G+C content [10]. LTA has a number of important functions in bacterial physiology including cation homeostasis, resistance to antimicrobial peptides, autolysin activity, non-covalent anchoring of cell-surface proteins, attachment to host tissues, and biofilm formation [1,11]. Glycolipids also function as acceptors of the glycerol-phosphate polymer during LTA synthesis, although the exact mechanism underlying this process is still under investigation [10]. If the processive glycosyltransferase YpfP is inactivated in *Staphylococcus aureus*, DAG instead of DGlcDAG is utilized as a building block in LTA synthesis, suggesting that glycolipids are not essential acceptors of the LTA polymer [12,13].

A second glycosyltransferase (EF 2890) is located immediately downstream of *bgsA*. To our knowledge, the function of this gene locus of *E. faecalis *or its homologues in streptococci is still unknown. In the current study, we report the construction of a deletion mutant of EF_2890 that we designated *bgsB *and studied the role of glycolipid metabolism in LTA biosynthesis and bacterial physiology.

## Results

### Construction of a deletion mutant of the glycosyltransferase *bgsB*

**Figure 1:**
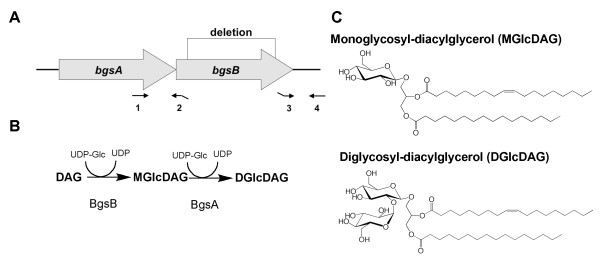
**Biosynthesis of glycolipids in *E. faecalis***. **A **Genetic organization of the *bgs*-locus in *E. faecalis*. The numbers refer to the primers described in Table 2. *bgsB *has a length of 1224 bp. A putative transcriptional terminator is found 10 bases downstream of *bgsB*. **B **Putative biosynthetic pathway of glycolipid synthesis in *E. faecalis*. **C **Structure of *E. faecalis *glycolipids. The position of 18:1 and 16:0 fatty acids has not been determined [5].

**Figure 2:**
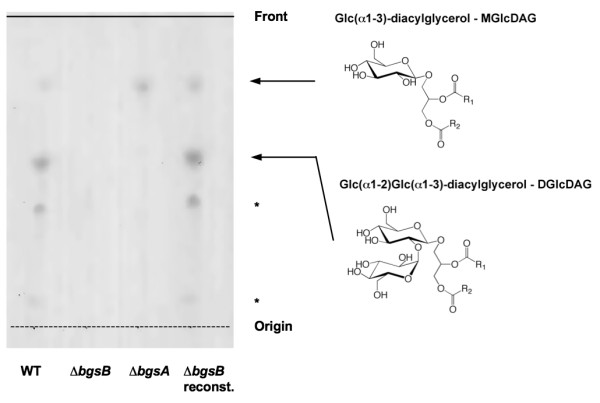
**Thin-layer chromatography of cell-membrane total lipid extracts of *E. faecalis *strains**. Bacterial cells were grown overnight, disintegrated, and stirred with butanol. Membrane lipids were extracted from butanol by phase partition according to Bligh and Dyer. 50 μg of lipid extract was applied to the TLC plate, separated using a solvent system of CHCl_3_/MeOH/H_2_O (65:25:4, v/v/v), and visualized with α-naphtol/sulfuric acid. The identity of the bands has been confirmed previously [5]. The glycolipids marked with an asterisk have not been analyzed.

**Figure 3:**
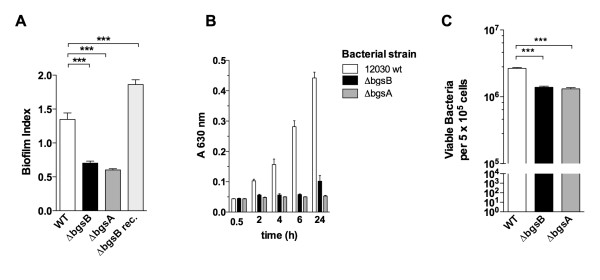
**Role of *bgsB *in biofilm formation and bacterial adherence to Caco-2 cells**. **A **Biofilm formation on polystyrene. Microtiter plates were incubated with bacteria for 18 h, non-adherent bacteria removed by washing with PBS, and biofilms stained with crystal violet. Data represent the means ± SEM. *** P < Tukey's multiple comparison test. **B **Development of biofilm on polystyrene of *E. faecalis *12030 wt, 12030Δ*bgsB*, and 12030Δ*bgsA *over time. After incubation periods of ≥ 4 h, *E. faecalis *12030 wt elaborated significantly more biofilm than the deletion mutants (P < 0.001, Tukey's multiple comparison test). Bars represent means ± SEM. **C **Bacterial adherence to Caco-2 cells. Caco-2 cells were incubated at a multiple of infection of 100:1 for 2 h with the respective strain grown to mid-log phase. Data represent the means ± SEM. *** P < 0.001, Dunn's multiple comparison test.

Immediately downstream from *bgsA*, we identified a putative 1,2-diacylglycerol 3-glucosyltransferase (TIGR number EF2890) by basic local alignment search tool (BLASTP) search (Figure 1). This glycosyl-transferase shows homology to YP_001620482.1 of *Acholeplasma laidlawii *(identity 34%, similarity 55%) [14] and to Lmo2555 of *Listeria monocytogenes *(identity 23%, similarity 41%) [15]. We designated this gene *bgsB*. To study the requirement of *bgsB *for glycolipid production, LTA synthesis, and bacterial physiology, we constructed a deletion mutant by targeted mutagenesis using the strategy previously applied for the *bgsA *deletion mutant. Unmarked deletions were created by allelic exchange, and all gene deletions were confirmed by PCR. In the resulting mutant, an internal fragment of 790 bp was deleted from the *bgsB *gene (Figure 1). Single gene reconstitution of *bgsB *in *E. faecalis *12030Δ*bgsB *completely restored the wild-type phenotype, including the glycolipid expression profile in cell membrane extracts (Figure 2) and biofilm formation (Figure 3).

### Deletion of *bgsB *leads to a complete loss of glycolipids from the cell membrane and to expression of LTA with increased chain length

We hypothesized that, because it is located immediately downstream from *bgsA *and has high homology to ALmgs in *Acholeplasma laidlawii*, the gene product of *bgsB *glycosylates diacylglycerol to yield MGlcDAG. To test this hypothesis, we extracted the total lipids of the cell membrane, separated them by thin layer chromatography (TLC), and stained glycolipids with α-naphthol (Figure 2). As shown previously, inactivation of *bgsA *resulted in accumulation of MGlcDAG in the cell membrane (Figure 2). In contrast, no glycolipids were visualized in 12030Δ*bgsB *extracts, suggesting that *bgsB *encodes for a glycosyltransferase that glycosylates DAG to form MGlcDAG. MGlcDAG is the substrate of BgsA, which adds a second glucose to yield DGlcDAG (Figure 1). Since BgsA does not accept DAG as a substrate, inactivation of BgsB results in the loss of all glycolipids from the cell membrane (Figure 2).

**Figure 4:**
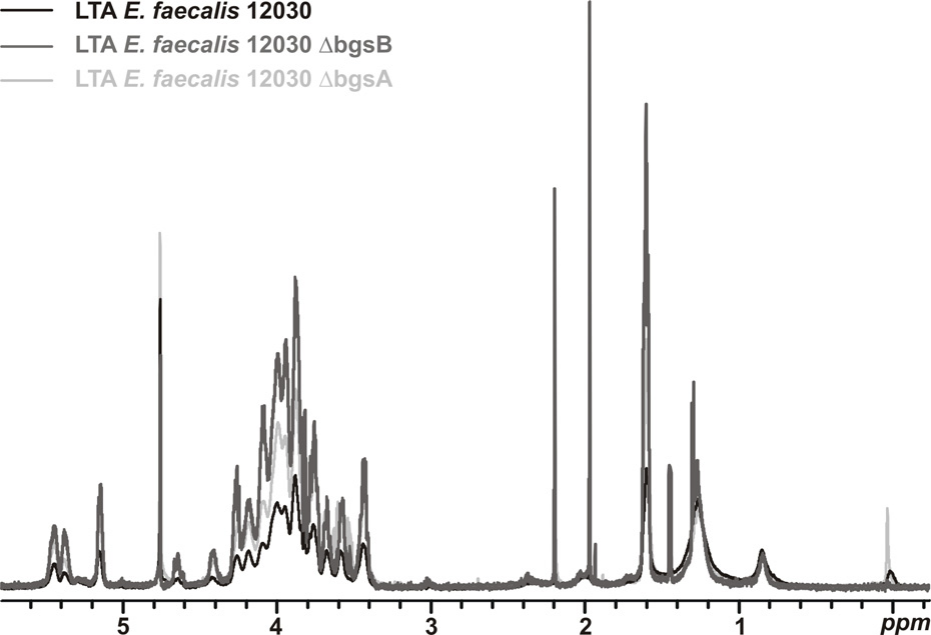
**Comparison of ^1^H-NMR spectra of LTA isolated from *E. faecalis *12030 wt, 12030Δ*bgsB*, and 12030Δ*bgsA***. Comparison of integration values of fatty acid (FA) signals (-CH_2_- and -CH_3_) as an internal reference and anomeric proton signal of glucose (H1 Glc A and H1 Glc B) indicated that the glycerolphosphate polymer of LTA from 12030Δ*bgsB *and 12030Δ*bgsA *contains approximately four times more kojibiose. Comparison of the resonance signal of total alanine (-CH_3 _Ala) and fatty acid signals (-CH2- (FA) and -CH3 (FA)) revealed that LTA extracted from either mutant also contains more alanine residues. Gro - glycerol.

**Figure 5:**
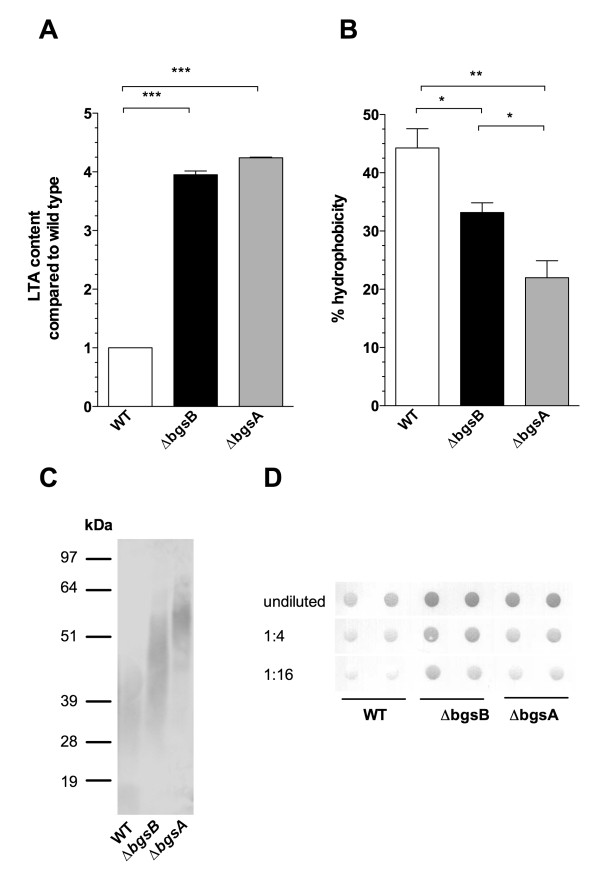
**Impact of *bgsB *on the synthesis and anchoring of LTA in the cell wall and on hydrophobicity of *E. faecalis *cells**. **A **The total amount of butanol-extracted LTA from cell-wall extracts as determined by ELISA. For the quantification of LTA tethered to the cell wall, bacteria were grown overnight and adjusted to the same OD_600_. Cell walls were disrupted by shaking with glass beads, and LTA was mobilized by stirring bacterial cells with butanol/water. ELISA plates were incubated with various concentrations of the respective water phase of the extraction, and LTA was detected using a polyclonal rabbit anti-LTA antibody. Data points represent means ± SEM, *** P < 0.001, Tukey's multiple comparison test. **B **Cell-surface hydrophobicity of *E. faecalis *strains determined by adherence of bacterial cells to a mixture of dodecane and aqueous phase. Bars represent the percentage of bacteria remaining in the organic phase after partitioning of the solvent system. Data represent the means ± SEM, **P < 0.01, *P < 0.05, Tukey's multiple comparison test. **C **Western blot detection of LTA from 12030 wild type and deletion mutants. LTA was extracted from disrupted bacterial cells after shaking with glass beads by boiling in SDS. LTA was loaded onto 4-12% SDS PAGE gels and detected using a polyclonal rabbit antibody against enterococcal LTA. **D **Estimation of LTA shed into the culture medium. After overnight culture, bacterial density was adjusted to the same OD_600_, and bacteria were removed by centrifugation. 100 μl of supernatant was blotted onto PVDF membrane. Bound LTA was detected using the same antibody used in the ELISA. Dilution steps of culture supernatant are indicated in the legend.

We recently showed that inactivation of *bgsA *also affects LTA synthesis, increasing the chain length of the glycerol-phosphate polymer [5]. Inactivation of *bgsB *has a similar effect on the LTA chain length (Figure 4). To estimate the chain length of the glycerol-phosphate chain by ^1^H-NMR analysis, we used the fatty acid signals of the molecule as an internal reference and compared the integration values of H1 of glucose and -CH3 of alanine to the -CH3 and -CH2- signals (δ 1.26-1.29, and 0.88) of the fatty acids [5]. The integral ratios yielded higher amounts of glucose and alanine incorporated into the LTA of 12030Δ*bgsB *and 12030Δ*bgsA *compared to the wild type, suggesting an increased length of the glycerol-phosphate polymer (Figure 4). These results are supported by quantification of LTA from butanol extracts by ELISA (Figure 5). Approximately fourfold more LTA was recovered from butanol extracts of cell walls of 12030Δ*bgsB *and 12030Δ*bgsA *than from wild-type bacteria. To determine whether increased amounts of LTA were also released into the culture medium, we blotted the culture supernatant onto PVDF membranes and performed semi-quantitative immuno-dot blot analysis (Figure 5). For both mutants, 12030Δ*bgsB *and 12030Δ*bgsA*, increased amounts of LTA in the liquid medium were detected, indicating a higher turnover of LTA in the cell envelope. Previous studies in *S. aureus *and *Listeria monocytogenes *have shown that substitution of DGlcDAG by MGlcDAG or DAG as the glycolipid anchor of LTA retards the migration of the molecule in SDS-PAGE [13,15]. LTA extracted from both mutants displayed a slower mobility in SDS PAGE than wild-type LTA, with LTA from 12030Δ*bgsB *migrating faster than LTA from 12030Δ*bgsA *(Figure 5). This suggests that both mutants express different lipid anchors from those in the wild type. As DAG is the only substrate available for LTA synthesis in 12030Δ*bgsB*, it likely serves as lipid anchor in this strain.

LTA and glycolipids are also major determinants of cell-surface charge density. Therefore, hydrophobicity of wild-type and mutant bacteria was determined by measuring the adherence to dodecane. Reduced adherence was observed for both 12030Δ*bgsA *and 12030Δ*bgsB *(Figure 5). However, 12030Δ*bgsB *had higher hydrophobicity than 12030Δ*bgsA *(44% wild type versus 33% 12030Δ*bgsB *and 22% 12030Δ*bgsA*).

### Bacterial physiology is not significantly impaired in a *bgsB *deletion mutant

Previous studies have shown that LTA and glycolipids play important roles in growth, cell envelope integrity, and cell division [11]. However, despite the complete lack of glycolipids in the cell membrane and increased production of LTA, important characteristics of 12030Δ*bgsB *did not differ from wild-type bacteria: Mutants did not differ from wild-type bacteria in their growth kinetics in broth culture (data not shown). Cell morphology of 12030Δ*bgsB *determined by transmission electron microscopy was not affected (Additional file 1). Likewise, autolysis was not affected in 12030Δ*bgsB *(Additional file 2). Since phosphatidylglycerol from the cell membrane is used as a substrate for polyglycerolphosphate synthesis by LtaS [10], we investigated whether increasing chain length of LTA affects cell membrane content of phosphatidylglycerol in the mutant. However, the semi-quantitative analysis of extracts of total membrane lipids by TLC and staining with molybdenum blue did not reveal differences in phospholipid composition (Additional file 3). The composition and total amount of aminophospholipids as assessed semi-quantitatively by TLC also did not differ between the wild type and 12030Δ*bgsB *(Additional file 3). Neither did analysis of non-covalently bound surface proteins by SDS-PAGE reveal major differences between the *bgsB *deletion mutant and the parental strain (Additional file 3).

### Deletion of the glucosyltransferase *bgsB *has no effect on resistance to complement, antimicrobial peptides, and opsonophagocytic killing

LTA has been shown to be critical for resistance against killing by cationic antimicrobial peptides [1] and has been identified as a target of opsonic antibodies against *E. faecalis *[4]. To characterize the sensitivity of 12030Δ*bgsB *to host defense mechanisms, we assessed its resistance to antimicrobial peptides nisin, polymyxin B, and colistin. For nisin, no difference was found between the wild-type and the *bgsB *deletion mutant (Additional file 4). A two-fold lower concentration of polymyxin B and colistin was required for killing of 12030Δ*bgsB *compared to the isogenic wild type strain. At a serum concentration of 1.7%, sensitivity to complement-mediated phagocytosis did not differ between the 12030 wild type and 12030Δ*bgsB *(Additional file 2). Furthermore, rabbit antibodies raised against whole bacterial cells of *E. faecalis *12030 mediated opsonophagocytic killing of 12030Δ*bgsB *comparable to levels obtained for the wild-type strain (Additional file 2).

### The loss of glycolipids from the cell membrane is associated with reduced adherence to Caco-2 cells and impaired biofilm formation

We recently showed that deletion of *bgsA *leads to loss of biofilm formation on polystyrene and to reduced adherence to Caco-2 cells [5]. Partial deletion of *bgsB *also strongly impaired biofilm formation, reducing production by 50% (Figure 3). This defect in biofilm formation was not a result of decreased initial attachment (i.e., bacteria attached in ≤ 30 min of incubation); rather, it was due to defective accumulation of biofilm mass after initial attachment (Figure 3). Over a period of 24 h, biofilm mass of wild-type bacteria on polystyrene grew in a linear fashion. In contrast, the amount of biofilm produced by *bgsB *and *bgsA *mutants remained constant at the level of initial attachment. Adhesion to colonic epithelial cells (Caco-2 cells) was also impaired in 12030Δ*bgsB*, reaching only 50% of the adhesion of wild-type bacteria (Figure 3).

### *bgsB *contributes to virulence during bacteremia in mice

**Figure 6:**
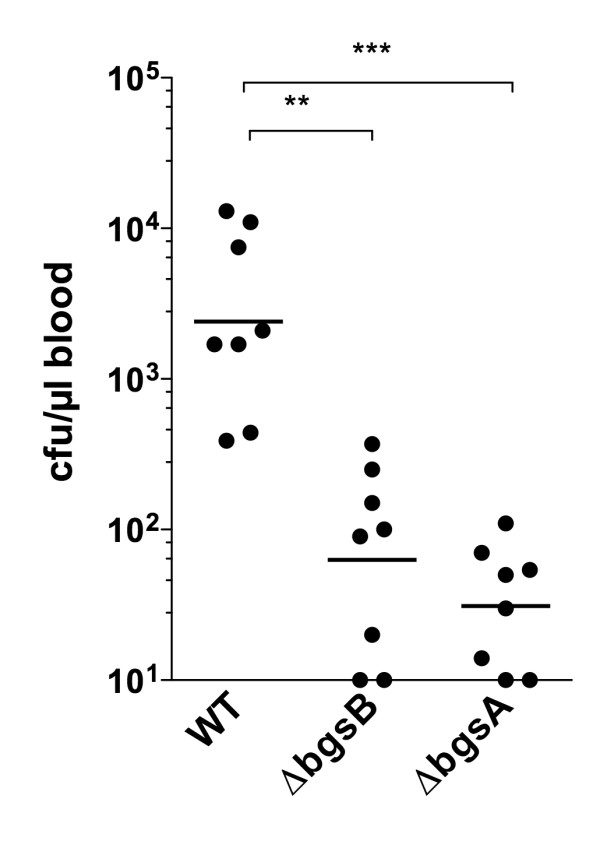
**Virulence of *E. faecalis*Δ*bgsB *in a mouse bacteremia model**. Female BALB/c mice 6-8 weeks old were infected via the tail vein with stationary-phase *E. faecalis *strains (2.0 × 10^9 ^cfu). After 72 h mice were sacrificed and bacterial counts in the blood were enumerated. Data represent the individual bacterial counts and the geometric mean. ** P < 0.01, *** P < 0.001, Dunn's multiple comparison test. The lower limit of detection of the assay was 10 CFU/ml blood.

Previous experiments with a *bgsA *deletion mutant in *E. faecalis *showed that it leads to an attenuation of virulence in a mouse bacteremia model [5]. To assess whether cell membrane glycolipids or glycolipid anchoring of LTA is required for the pathogenesis of enterococcal infections, we employed the same model to investigate the *bgsB *mutant. As mentioned above, 12030 wild-type and respective mutants had comparable growth characteristics. For virulence studies, we infected BALB/c mice 6 - 8 weeks old by i.v. injection, sacrificed the animals after 3 days, and enumerated the viable bacteria. Pilot experiments indicated that, with a high inoculum of 2 × 10^9 ^bacteria, infected mice are bacteremic up to 4 days without succumbing to the infection. Compared to the wild type, mice infected with 12030Δ*bgsB *or 12030Δ*bgsA *cleared significantly more bacteria from the bloodstream (Figure 6). No difference in virulence between 12030Δ*bgsB *and 12030Δ*bgsA *was detected in this model.

## Discussion

Although MGlcDAG and DGlcDAG are the major glycolipids in many Gram-positive bacteria, distinct differences are found between species in biosynthetic pathways. In staphylococci and *Bacillus*, a single processive glucosyltransferase YpfP adds two glucose residues to DAG to synthesize DGlcDAG [12,16,17]. Depending on the bacterial species and strain background, the deletion of this enzyme may result in an increased LTA content and turnover [16], or loss of LTA from the cell membrane, associated with a reduced rate of autolysis and impaired biofilm formation [12]. In listeria, streptococci, and enterococci, genome analysis revealed two putative glycosyltransferases involved in the biosynthetic pathway of glycolipids [7,14,15,18]. Homologues of a (1→2) glucosyltransferase have been investigated in listeria (LafA), group B streptococci (IagA), and *E. faecalis *(BgsA) [5,15,18]. In group B streptococci, deletion of *iagA *results in the absence of capsule expression, reduced retention of LTA on the bacterial cell surface, and increased release of LTA into the culture medium [18]. Inactivation of *lafA *in *L. monocytogenes *strongly depletes LTA from both the cell wall and the culture medium [18]. In contrast to these findings, deletion of *bgsA *in *E. faecalis *results in an increased concentration of LTA in the bacterial cell envelope, most likely related to the longer glycerol-phosphate polymer. The different makeup of glycolipids and LTA in this mutant strongly impaired biofilm-formation and affected virulence in vivo [5].

In the current study, we constructed a deletion mutant by targeted mutagenesis of the putative glycosyltransferase *bgsB *located immediately downstream of *bgsA*. After inactivation of *bgsB *in *E. faecalis *12030, no glycolipids or glycolipid-derivatives were recovered from the cell envelope of the 12030Δ*bgsB *mutant, indicating that BgsB is a 1,2-diacylglycerol 3-glucosyltransferase. BgsA cannot take the place of BgsB, which suggests that BgsA has higher substrate specificity than YpfP in *S. aureus *and *B. subtilis *[13,17]. The putative function assigned to BgsA and BgsB by this work is in agreement with data obtained for their homologues LafA and LafB in *L. monocytogenes *[15]. Although the lipid anchor of LTA from 12030Δ*bgsB *was not characterized chemically, indirect evidence suggests that DAG instead of DGlcDAG anchors LTA to the cell membrane in this mutant. LTA extracted from 12030Δ*bgsB *migrated more slowly than wild-type LTA in SDS PAGE, a feature that has been described for homologous LTA molecules substituted with DAG instead of DGlcDAG in *S. aureus *and *L. monocytogenes *[13,15]. In staphylococci and listeria it has been also demonstrated that, in the absence of glycolipids, the enzyme that transfers glycerolphosphate residues to the glycolipid anchor (LtaS) can utilize DAG as glycerolphosphate acceptor for the synthesis of the LTA backbone [13,15]. Deletion mutants of the glucosyltransferases *bgsB *and *bgsA *enabled us to study the individual roles of the two major glycolipids MGlcDAG and DGlcDAG in the physiology and virulence of *E. faecalis*. To our surprise, the complete loss of glycolipids from the cell membrane in 12030Δ*bgsB *had only minor effects on bacterial morphology, cell growth, and autolysis.

In contrast, MGlcDAG and DGlcDAG are critical for cell membrane elasticity and fluidity and important for the function of membrane-bound proteins in *Acholeplasma laidlawii *[6,7,14]. It is possible, however, that up-regulation of other cell membrane amphiphiles may compensate for the lack of glycolipids in the *bgsB *mutant [6]. In fact, the concentration of LTA was increased in 12030Δ*bgsB *and possibly compensates for the loss of phosphoglycolipid derivatives of MGlcDAG and DGlcDAG in the 12030Δ*bgsB *mutant [19]. A characteristic feature of both mutants is the increased chain length of the glycerol-phosphate polymer. However, the mechanism underlying this alteration in LTA structure remains unclear and deserves further attention.

The most notable feature of 12030Δ*bgsB *is its impairment in biofilm formation and adherence to colonic cells. As observed previously in the *bgsA *mutant, initial attachment to polystyrene was not impaired in 12030Δ*bgsB*, but the accumulation of bacteria in the growing biofilm was impaired. This is in contrast to other biofilm-defective mutants in *E. faecalis*, in which attachment to the foreign surface is the feature primarily affected and underlines the importance of cell envelope amphiphiles in the retention of bacteria within the biofilm architecture [20,21]. Several mechanisms may explain the biofilm phenotype of the mutants. As in the *bgsA *mutant, impaired biofilm formation in 12030Δ*bgsB *was associated with reduced hydrophobicity, a well-known determinant of biofilm formation in bacteria [22,23]. Also, increased LTA concentration in the cell envelope of the *bgsB*-mutant may impair biofilm formation by increasing the net negative charge of the cell envelope. The impact of the higher negative charge of the LTA molecule on biofilm formation has been demonstrated by mutants in the D-alanine-D-alanyl-carrier protein ligase DltA [24,25]. Finally, the increased amount of LTA released into the biofilm matrix (as observed with 12030Δ*bgsB *and 12030Δ*bgsA*) may act as a biosurfactant, promoting detachment of bacterial cells from the biofilm and thereby impeding its growth [26]. In contrast to our results the inactivation of the glycosyltransferase YpfP in *S. aureus *leads to depletion of LTA from the cell surface and to a reduced ability to form biofilm [12].

Aside from its effects on biofilm formation, the increased density of negative charges of the LTA molecule of the mutant may also explain the slight increase in sensitivity of 12030Δ*bgsB *to the antimicrobial peptides colistin and polymyxin B. If this difference explains the significantly impaired virulence in our mouse bacteremia model, however, is unclear. On balance, we observed a 2-log reduction in the number of CFU recovered for both mutants, suggesting that glycolipids, either as a cell membrane component or as an anchor of LTA, play a critical role in the cell envelope of enterococci during infection. In general, mutation of the glycosyl-transferase *bgsA *and *bgsB *yielded similar phenotypes, suggesting that the phenotypic changes observed for both mutants are mainly the result of the depletion of DGlcDAG or altered LTA structure. On the other hand, MGlcDAG seems to play a minor role in bacterial physiology and virulence.

## Conclusions

We have shown that the *bgsB *gene is responsible for the glycosylation of DAG to form MGlcDAG, the first step in glycolipid synthesis in *E. faecalis*. *bgsB *deletion led to reduced biofilm formation and attachment to colonic cells, and to impaired virulence in vivo.

## Methods

### Bacterial strains, plasmids, and growth conditions

The bacterial strains and plasmids used in this study are shown in Table 1. Enterococci were grown at 37°C without agitation in tryptic soy broth (TSB; Merck), M17 broth (Difco Laboratories), or TSB plus 1% glucose (TSBG) as indicated. In addition, tryptic soy agar or M17 agar plates were used. *Escherichia coli *DH5α and TOP10 (Invitrogen) were cultivated aerobically in LB-broth. Kanamycin was added for enterococci (1 mg/ml) and for *E. coli *(50 μg/ml); tetracycline was used at 12.5 μg/ml for *E. coli *and at 10 μg/ml for enterococci.

**Table 1:** *E. faecalis *strains and plasmids used in this study.

strain or plasmid	characterization	reference
**strains**		

*E. faecalis *12030	Clinical isolate, strong biofilm producer	[33]

*E. faecalis *ATCC 29212	Reference strain	

*E. faecalis *12030Δ*bgsA*	(EF2891) *bsgA *mutant	[5]

*E. faecalis *12030Δ*bgsB*	*bgsB *deletion mutant	This study

*E. faecalis *12030Δ*bgsB_rec*.	Reconstituted mutant	This study

*Escherichia coli *DH5α	Gram-negative cloning host	

*Escherichia coli *TOP10	Gram-negative cloning host	Invitrogen

		

**plasmids**		

pCASPER	Gram-positive, temperature-sensitive mutagenesis vector	[34]

pCRII-TOPO	Gram-negative cloning vector	Invitrogen

pCASPER/Δ*bgsB*		This study

pMAD/*bsg*B		This study

pMAD	oripE194^ts^, Em^R^, Amp^R^, *bgaB*	[35]

### Construction of a nonpolar deletion mutant of *bgsB*

Molecular techniques used in this study have been described previously [5]. In brief, the *bgsB *mutant was constructed in *E. faecalis *12030 by homologous recombination. The deletion of a portion of the gene *bgsB *(790 bp) (EF_2890 in the *E. faecalis *V583 genome, GenBank accession no. AAO82579.1) was created as described elsewhere [5]. Primers 1 and 2 (Table 2) were used to amplify a 581-bp fragment downstream, and primers 3 and 4 were used to amplify a 563-bp fragment upstream of the target gene. Primers 2 and 3 contain a 21-bp complementary sequence (underlined in Table 2). Overlap extension PCR was performed to generate a PCR product lacking a fragment of 790 bp in the center of *bgsB *(Figure 1). The resulting construct was cloned into the Gram-positive shuttle vector pCASPER containing a temperature-sensitive replicon; the resulting plasmid, pCASPER-Δ*bgsB*, was transformed into *E. faecalis *12030 by electroporation. Integrants were selected at the non-permissive temperature (42°C) on TSA plates with kanamycin. Colonies were passaged between 6 and 10 times in liquid cultures without antibiotics at the permissive temperature (30°C) and subsequently screened by replica-plating for loss of kanamycin resistance. Kanamycin-sensitive clones were analyzed by PCR for the deleted sequence, and the deletion mutant was designated *E. faecalis *12030Δ*bgsB*.

**Table 2:** Primers used in this study.

	Name	Sequence (5'-3')
1	EF2890 delF	CAAACTGCTCCTTCAGCAACT

2	EF2890 OEL	ACTAGCGCGGCCGCTTGCTCCCTATTTTGTCAGCGCCTCAAC

3	EF2890 OER	GGAGCAAGCGGCCGCGCTAGTTAGAAGTCGCTACCCCACTCA

4	EF2890 delR	GCGCGACAGTTACCAGAGTAT

Complementation of the 12030Δ*bgsB *mutant has been done by a knocking in strategy as described previously [27]. Briefly, the *bgsB *gene (1224 bp) plus 212 bp upstream and 502 bp downstream was amplified using primers 1 and 4, cloned into pCRII-TOPO (Table 1) and digested with *Eco*RI. The resulting fragment was inserted into plasmid pMAD (Table 1). *E. faecalis *12030Δ*bgsB *was transformed with the recombinant plasmid (pMAD-bgsB) and incubated at 37°C for 4 d on TSB plates supplemented with Xgal (40 μg/ml) and erythromycin (Erm, 100 μg/ml). Dark blue colonies were picked and incubated overnight on fresh plates supplemented with Xgal and Erm at the non-permissive temperature (44°C). Presence of the wild-type and mutated alleles was determined by PCR, and for each construct the positive clones were cultured in TSB medium supplemented with Erm (150 μg/ml) at 44°C over-night. This last step was repeated once, using the overnight culture to inoculate a fresh culture tube. To delete the erythromycin resistance gene, overnight cultures were inoculated in TSB medium without Erm and incubated for 12 h at 30°C, followed by 18 h at 44°C without shaking. This step was repeated until white colonies were obtained on Xgal-supplemented TSA plates incubated overnight at 37°C. Erm sensitivity of the white colonies was verified, and sensitive clones were tested by PCR for the presence of the intact *bgsB *gene.

### Biofilm plate assay

Enterococci were tested for production of biofilm using a polystyrene microtiter assay [5,24]. Briefly, bacteria were grown at 37°C in TSB for 18 h. Polystyrene tissue-culture plates (Brandt, Germany) were filled with 180 μl of TSB plus 1% glucose and 20 μl of this culture, and the plates were then incubated at 37°C for 18 h. The plates were read in an ELISA reader (Bio-Rad Microplate reader) at an optical density of 630 nm to assess homogenous growth. The culture medium was discarded, and the wells were washed 3 times with 200 μl of PBS without disturbing the biofilm on the bottom of the wells. The plates were dried at 60°C for 1 h and stained with 2% Hucker's crystal violet for 2 min. Excess stain was removed by rinsing the plates under tap water, and the plates were dried at 60°C for 10 min. The optical density at 630 nm was determined. Biofilm formation was normalized to growth with the biofilm index, which was calculated as OD of the biofilm × (0.5/OD of growth) [24].

### Adherence to Caco-2 cells

Adherence to Caco-2 cells was investigated using methods described previously [28]. In brief, cells were cultivated in DMEM medium supplemented with 10% fetal bovine serum and 1% non-essential amino acids under a 5% CO_2 _atmosphere. All the experiments were performed on cells between the 15^th ^and 25^th ^passage. Caco-2 cells were cultivated in 24-well plates to a density of 1 × 10^5 ^cells/well for 3-5 days. Bacteria were grown to mid-log phase at 37°C without agitation in tryptic soy broth; Caco-2 cells were incubated with bacteria for 2 h at a multiplicity of infection of 100:1. After infection of the monolayer, epithelial cells were washed and lysed with 0.25% Triton-X at 37°C for 20 min and adherent bacteria enumerated by quantitative bacterial counts. Pilot experiments had shown no significant bacterial invasion under the outlined conditions.

### Isolation and analysis of glycolipids and LTA

Bacterial cells were resuspended in 0.1 M citrate buffer pH 4.7 and cell walls disrupted by shaking with an equal volume of glass beads (0.1 mm glass beads, 3 × 1 min intervals using a BeadBeater, Glenn Mills, Clifton, NJ). Glass beads were removed by sedimentation, and disrupted cells were stirred with an equal volume of *n*-butanol for 30 min. After phase separation by centrifugation, the aqueous layer was removed, dialyzed against 0.1 M ammonium acetate (pH 4.7) and lyophilized. LTA was purified from the aqueous phase by hydrophobic interaction chromatography [4]. The butanol phase was evaporated under a vacuum, and cell membrane lipids were extracted according to the method of Bligh and Dyer and separated by TLC (0.2 mm Silica gel 60 F_254 _Merck, Darmstadt) using a solvent system of CHCl_3_/MeOH/H_2_O (65:25:4, v/v/v) and detection with α-naphthol (3.2%). For detection of phospholipids, TLC plates were stained with molybdenum blue; amino phospholipids were stained with ninhydrin, as previously described [29]. LTA was also analyzed by SDS-PAGE as described previously [5]. Briefly, bacterial cell walls were disrupted by shaking with glass beads as described above, boiled in sample buffer containing SDS, and subjected to SDS-PAGE in gradient gels containing acrylamide (4/12% w/v, Invitrogen). Separated LTA was transferred onto PVDF membrane and blocked at 4°C in Tris-buffered saline (TBS) containing skim milk (5% w/v) for 18 h, then incubated at 20-22°C for 2 h with rabbit antibody raised against *E. faecalis *LTA (see below) diluted 1:200 in TBS/skim milk. After washing in TTBS (Tween 20 0.05% v/v in TBS), the sheets were incubated at 20-22°C for 1 h with a goat anti-rabbit IgG (whole cell) alkaline phosphatase conjugate (Sigma), diluted 1:1000 with TBS/skim milk, and then washed again in TTBS. Binding of the enzyme-conjugated antibodies was detected with the NBI/BCIP (Biorad). For visualization of proteins, SDS PAGE gels were stained with Coomassie blue.

### Characterization of LTA

Lipoteichoic acid of *E. faecalis *strain 12030Δ*bgsB *was analyzed by NMR spectroscopy as described previously [5].

### Rabbit antiserum against LTA

A female New Zealand White rabbit was immunized s.c. with 100 mg of LTA purified from *E. faecalis *strain 12030 suspended in complete Freund adjuvant (Sigma), followed by the same dose s.c. suspended in incomplete Freund adjuvant (Sigma) on day 7. The rabbit was boosted intravenously with three 10-mg doses over the following 3 weeks. After the last vaccination, the rabbit was sacrificed and exsanguinated to obtain the serum.

### Autolysis assay and sensitivity to antimicrobial peptides

Cell autolysis was determined as described by Qin et al. [30]. The MIC of polymyxin B, nisin, and colistin against wild-type and 12030Δ*bgsB *were determined by a modified NCCLS broth dilution method [24].

### Determination of hydrophobicity

Hydrophobicity was determined by measuring adherence to dodecane [31]. Briefly, bacteria were grown to logarithmic phase and resuspended in sodium phosphate to yield an OD_600 _of 0.4-0.5. The same volume of dodecane was added, and phases were vigorously vortexed for 1 min, then for 10 min to allow phase separation. Absorbance of the water-phase was measured. The proportion of cells in the dodecane phase was calculated according to the formula: % hydrophobicity = [1-(A/A0)] × 100.

### Mouse bacteremia model

The virulence of *E. faecalis *strain 12030Δ*bgsB *was evaluated in a mouse bacteremia model [5,32]. In summary, eight female BALB/c mice 6-8 weeks old were challenged by i.v. injection of *E. faecalis *strains grown to stationary phase (2.0 × 10^9 ^cfu) via the tail vein. Seventy-two hours after infection, the mice were sacrificed and exsanguinated, and bacterial counts in the blood were enumerated by serial dilutions. All animal experiments were performed in compliance with the German animal protection law (TierSchG). The mice were housed and handled in accordance with good animal practice as defined by FELASA and the national animal welfare body GV-SOLAS. The animal welfare committees of the University of Freiburg (Regierungspräsidium Freiburg Az 35/9185.81/G-07/15) approved all animal experiments.

### Transmission electron microscopy (TEM)

Bacterial cells were prepared for TEM as described previously [24].

### Opsonophagocytic killing assay

An opsonophagocytic killing assay was used as previously described [5]. In summary, white blood cells (WBC) were prepared from fresh human blood collected from healthy adult volunteers. Using trypan blue staining to differentiate dead from live leukocytes, the final cell count was adjusted to 2.5 × 10^7 ^WBC per ml. Baby rabbit serum (Cedarlane Laboratories, Hornby, Ontario, Canada), diluted 1:15 in RPMI plus 15% fetal bovine serum (FBS) and absorbed with the target strain, was used as complement source. Bacteria cultured on agar plates were resuspended in TSB to an OD_600 _of 0.1 and then grown to an OD of 0.4. A final 1:100 dilution was made in RPMI-FBS. Equal amounts of PMN and bacteria (multiple of infection 1:1), complement, and heat-inactivated rabbit immune serum were incubated at 37°C for 90 min. For the controls, antibody, complement, or PMN were replaced by RPMI-FBS. For enumeration of surviving bacteria, the content of tubes was diluted in TSB, and samples were plated onto tryptic soy agar plates. The percentage of opsonophagocytic killing was calculated by determining the ratio of the CFU surviving in the tubes with bacteria, leukocytes, complement, and antibody to the CFU surviving in the tubes with all these components but lacking leukocytes.

### Quantification of LTA

The LTA content of bacterial cell walls was measured according to the method of Fedtke et al. [12]. In summary, wild-type and mutant bacteria were grown for 18 h in TSB, adjusted to an equal OD_600_, and bacteria from equal volumes were collected by centrifugation. Bacterial were disrupted by shaking with glass beads as described above, and LTA was extracted from the cell walls by stirring them in an equal volume of butanol and 0.1 M Na-acetate buffer (pH 4,7). The aqueous phase of the extract was dialyzed, lyophilized, and resuspended in the same volume of phosphate buffer (pH 7.0). ELISA plates (Brandt) were coated with a range of LTA dilutions at 4°C for 18 h, and adherent LTA was detected using a rabbit antiserum specific for *E. faecalis *LTA as primary antibody (see above). A goat anti-rabbit IgG whole-molecule alkaline phosphatase conjugate (Sigma) served as secondary antibody [5]. LTA from *E. faecalis *12030, purified by hydrophobic-interaction chromatography, was used as a standard. The amount of LTA shed into the culture medium was measured semi-quantitatively by immuno-dot-blot analysis. To this end, bacteria were grown in TSB at 37°C for 18 h and adjusted to the same OD_600_. Bacterial cells were removed by centrifugation, culture supernatant was passed through a 0.45 μm membrane filter, and 100 μl of supernatant was spotted in various dilutions onto PVDF membrane using a dot-blot microfiltration apparatus (Bio-Dot, Biorad Laboratories, Munich, Germany). The membranes were allowed to dry overnight. Staining of immuno-dot-blots was performed using the same protocol as described for western blot analysis of LTA.

### Statistical Methods

Comparisons were made by one-way ANOVA and Tukey's multiple comparison test (parametric data) or Kruskal-Wallis test and Dunn's multiple comparison test (nonparametric data) as indicated using the Prism Graphpad 4 software package. A p-value of < 0.05 was considered statistically significant.

## Authors' contributions

CT participated in the isolation and TLC analysis of glycolipids and LTA, the design and interpretation of the experiments, made the statistical analysis, and drafted the manuscript. IS performed the cell culture assays, autolysis assay and hydrophobicity assay. YB carried out the biofilm assay and participated in the molecular genetic studies. AK performed the opsonophagocytic killing assay and the mouse infection model. PSC performed the biochemical analysis of glycolipids and LTA. EG participated in the draft of the manuscript. OH participated in the biochemical analysis of the glycolipids and LTA and the draft of manuscript. JH participated in the design, coordination and interpretation of the study, and the draft of the manuscript. All authors read and approved the final manuscript.

## Supplementary Material

Additional file 1 **Transmission electron microscopy of *E. faecalis *strains**. *E. faecalis *12030 wild type (A) and 12030Δ*bgsB *(B). Bar represents 500 nm. Click here for file

Additional file 2 **Autolysis and opsonization of *E. faecalis *12030Δ*bgsB***. **A **Spontaneous bacterial autolysis. Cells were grown to mid-log phase, resuspended in 10 mM sodium phosphate buffer containing 5% Triton X-100 and the decrease of the OD 600 at 30°C was recorded over time. **B **Bacterial killing in vitro after 90 min in the presence of 6.5% rabbit complement (white bar), 2 × 10^7 ^human PMN plus complement (gray bar) and rabbit antiserum raised against whole bacterial cells (serum dilution 1:2500) plus PMN and complement (black bar). Bars represent means ± SEM. Click here for file

Additional file 3 **Characterization of *E. faecalis *Δ*bgsB *cell walls**. **A **Thin-layer chromatography of cell membrane total lipid extracts of *E. faecalis *12030 wild type (lane 1 and 4), 12030Δ*bgsB *(lane 2 and 5), 12030Δ*bgsA *(lane 3 and 6). TLC plates were developed using a solvent system of CHCl_3_/MeOH/H_2_0 (65:25:4, v/v/v). Staining lane 1 - 3 molybdenum blue, lane 4 - 6 ninhydrin. **B **SDS PAGE of bacterial whole protein extracts. The material was extracted by disrupting the cells with glass-beads, boiling in Laemmli buffer, separated by 4-12% Bis-Tris gels and stained with Coomassie blue. Click here for file

Additional file 4 **Minimal bactericial concentration of *E. faecalis *strains against antimicrobial peptides**. Concentrations are expressed as μg/ml. Click here for file

## References

[B1] Weidenmaier C Peschel A Teichoic acids and related cell-wall glycopolymers in Gram-positive physiology and host interactions Nat Rev Microbiol 2008 6 4 276 287 10.1038/nrmicro1861 18327271

[B2] Theilacker C Kaczynski Z Kropec A Sava I Ye L Bychowska A Holst O Huebner J Serodiversity of Opsonic Antibodies against Enterococcus faecalis -Glycans of the Cell Wall Revisited PLoS ONE 2011 6 3 e17839 10.1371/journal.pone.0017839 21437253 PMC3060912

[B3] Teng F Singh KV Bourgogne A Zeng J Murray BE Further characterization of the epa gene cluster and Epa polysaccharides of Enterococcus faecalis Infect Immun 2009 77 9 3759 3767 10.1128/IAI.00149-09 19581393 PMC2737988

[B4] Theilacker C Kaczynski Z Kropec A Fabretti F Sange T Holst O Huebner J Opsonic antibodies to Enterococcus faecalis strain 12030 are directed against lipoteichoic acid Infect Immun 2006 74 10 5703 5712 10.1128/IAI.00570-06 16988246 PMC1594888

[B5] Theilacker C Sanchez-Carballo P Toma I Fabretti F Sava I Kropec A Holst O Huebner J Glycolipids are involved in biofilm accumulation and prolonged bacteraemia in Enterococcus faecalis Mol Microbiol 2009 71 4 1055 1069 10.1111/j.1365-2958.2008.06587.x 19170884

[B6] Wikström M Xie J Bogdanov M Mileykovskaya E Heacock P Wieslander A Dowhan W Monoglucosyldiacylglycerol, a foreign lipid, can substitute for phosphatidylethanolamine in essential membrane-associated functions in Escherichia coli J Biol Chem 2004 279 11 10484 10493 14688287 10.1074/jbc.M310183200

[B7] Edman M Berg S Storm P Wikstrom M Vikstrom S Ohman A Wieslander A Structural features of glycosyltransferases synthesizing major bilayer and nonbilayer-prone membrane lipids in Acholeplasma laidlawii and Streptococcus pneumoniae J Biol Chem 2003 278 10 8420 8428 10.1074/jbc.M211492200 12464611

[B8] Vikström S Li L Wieslander A The nonbilayer/bilayer lipid balance in membranes. Regulatory enzyme in Acholeplasma laidlawii is stimulated by metabolic phosphates, activator phospholipids, and double-stranded DNA J Biol Chem 2000 275 13 9296 9302 10734070 10.1074/jbc.275.13.9296

[B9] Campbell J Davies G Bulone V Henrissat B A classification of nucleotide-diphospho-sugar glycosyltransferases based on amino acid sequence similarities Biochem J 1998 329 Pt 3 719 9445404 10.1042/bj3290719 PMC1219098

[B10] Rahman O Dover LG Sutcliffe IC Lipoteichoic acid biosynthesis: two steps forwards, one step sideways? Trends Microbiol 2009 17 6 219 225 10.1016/j.tim.2009.03.003 19464183

[B11] Neuhaus FC Baddiley J A continuum of anionic charge: structures and functions of D-alanyl-teichoic acids in gram-positive bacteria Microbiol Mol Biol Rev 2003 67 4 686 723 10.1128/MMBR.67.4.686-723.2003 14665680 PMC309049

[B12] Fedtke I Mader D Kohler T Moll H Nicholson G Biswas R Henseler K Götz F Zähringer U Peschel A A Staphylococcus aureus ypfP mutant with strongly reduced lipoteichoic acid (LTA) content: LTA governs bacterial surface properties and autolysin activity Mol Microbiol 2007 65 4 1078 1091 10.1111/j.1365-2958.2007.05854.x 17640274 PMC2169524

[B13] Grundling A Schneewind O Genes required for glycolipid synthesis and lipoteichoic acid anchoring in Staphylococcus aureus J Bacteriol 2007 189 6 2521 2530 10.1128/JB.01683-06 17209021 PMC1899383

[B14] Berg S Edman M Li L Wikstrom M Wieslander A Sequence properties of the 1,2-diacylglycerol 3-glucosyltransferase from Acholeplasma laidlawii membranes. Recognition of a large group of lipid glycosyltransferases in eubacteria and archaea J Biol Chem 2001 276 25 22056 22063 10.1074/jbc.M102576200 11294844

[B15] Webb AJ Karatsa-Dodgson M Grundling A Two-enzyme systems for glycolipid and polyglycerolphosphate lipoteichoic acid synthesis in Listeria monocytogenes Mol Microbiol 2009 74 2 299 314 10.1111/j.1365-2958.2009.06829.x 19682249 PMC2764115

[B16] Kiriukhin MY Debabov DV Shinabarger DL Neuhaus FC Biosynthesis of the glycolipid anchor in lipoteichoic acid of Staphylococcus aureus RN4220: role of YpfP, the diglucosyldiacylglycerol synthase J Bacteriol 2001 183 11 3506 3514 10.1128/JB.183.11.3506-3514.2001 11344159 PMC99649

[B17] Jorasch P Wolter FP Zähringer U Heinz E A UDP glucosyltransferase from Bacillus subtilis successively transfers up to four glucose residues to 1,2-diacylglycerol: expression of ypfP in Escherichia coli and structural analysis of its reaction products Mol Microbiol 1998 29 2 419 430 10.1046/j.1365-2958.1998.00930.x 9720862

[B18] Doran KS Engelson EJ Khosravi A Maisey HC Fedtke I Equils O Michelsen KS Arditi M Peschel A Nizet V Blood-brain barrier invasion by group B Streptococcus depends upon proper cell-surface anchoring of lipoteichoic acid J Clin Invest 2005 115 9 2499 2507 10.1172/JCI23829 16138192 PMC1193870

[B19] Fischer W Hanahan DJ Bacterial phosphoglycolipids and lipoteichoic acids Handbook of Lipid Research 1990 6 New York: Plenum Press 123 234

[B20] Mohamed JA Huang DB Biofilm formation by enterococci J Med Microbiol 2007 56 Pt 12 1581 1588 10.1099/jmm.0.47331-0 18033823

[B21] Mohamed JA Huang W Nallapareddy SR Teng F Murray BE Influence of origin of isolates, especially endocarditis isolates, and various genes on biofilm formation by Enterococcus faecalis Infect Immun 2004 72 6 3658 3663 10.1128/IAI.72.6.3658-3663.2004 15155680 PMC415661

[B22] Bos R van der Mei HC Busscher HJ Physico-chemistry of initial microbial adhesive interactions--its mechanisms and methods for study FEMS Microbiol Rev 1999 23 2 179 230 10234844 10.1111/j.1574-6976.1999.tb00396.x

[B23] Courtney HS Ofek I Penfound T Nizet V Pence MA Kreikemeyer B Podbielski A Podbielbski A Hasty DL Dale JB Relationship between expression of the family of M proteins and lipoteichoic acid to hydrophobicity and biofilm formation in Streptococcus pyogenes PLoS ONE 2009 4 1 e4166 10.1371/journal.pone.0004166 19132104 PMC2613554

[B24] Fabretti F Theilacker C Baldassarri L Kaczynski Z Kropec A Holst O Huebner J Alanine esters of enterococcal lipoteichoic acid play a role in biofilm formation and resistance to antimicrobial peptides Infect Immun 2006 74 7 4164 4171 10.1128/IAI.00111-06 16790791 PMC1489678

[B25] Gross M Cramton SE Götz F Peschel A Key role of teichoic acid net charge in Staphylococcus aureus colonization of artificial surfaces Infect Immun 2001 69 5 3423 3426 10.1128/IAI.69.5.3423-3426.2001 11292767 PMC98303

[B26] Neu TR Significance of bacterial surface-active compounds in interaction of bacteria with interfaces Microbiol Rev 1996 60 1 151 166 8852899 10.1128/mr.60.1.151-166.1996 PMC239423

[B27] La Carbona S Sauvageot N Giard JC Benachour A Posteraro B Auffray Y Sanguinetti M Hartke A Comparative study of the physiological roles of three peroxidases (NADH peroxidase, Alkyl hydroperoxide reductase and Thiol peroxidase) in oxidative stress response, survival inside macrophages and virulence of Enterococcus faecalis Mol Microbiol 2007 66 5 1148 1163 10.1111/j.1365-2958.2007.05987.x 17971082

[B28] Sava IG Zhang F Toma I Theilacker C Li B Baumert TF Holst O Linhardt RJ Huebner J Novel interactions of glycosaminoglycans and bacterial glycolipids mediate binding of enterococci to human cells J Biol Chem 2009 284 27 18194 18201 10.1074/jbc.M901460200 19395379 PMC2709396

[B29] Peschel A Jack RW Otto M Collins LV Staubitz P Nicholson G Kalbacher H Nieuwenhuizen WF Jung G Tarkowski A Staphylococcus aureus resistance to human defensins and evasion of neutrophil killing via the novel virulence factor MprF is based on modification of membrane lipids with l-lysine J Exp Med 2001 193 9 1067 1076 10.1084/jem.193.9.1067 11342591 PMC2193429

[B30] Qin X Singh KV Xu Y Weinstock GM Murray BE Effect of disruption of a gene encoding an autolysin of Enterococcus faecalis OG1RF Antimicrob Agents Chemother 1998 42 11 2883 2888 9797220 10.1128/aac.42.11.2883 PMC105960

[B31] Reid G Cuperus PL Bruce AW van der Mei HC Tomeczek L Khoury AH Busscher HJ Comparison of contact angles and adhesion to hexadecane of urogenital, dairy, and poultry lactobacilli: effect of serial culture passages Appl Environ Microbiol 1992 58 5 1549 1553 1622224 10.1128/aem.58.5.1549-1553.1992 PMC195639

[B32] Hufnagel M Koch S Creti R Baldassarri L Huebner J A putative sugar-binding transcriptional regulator in a novel gene locus in Enterococcus faecalis contributes to production of biofilm and prolonged bacteremia in mice J Infect Dis 2004 189 3 420 430 10.1086/381150 14745699

[B33] Huebner J Wang Y Krueger WA Madoff LC Martirosian G Boisot S Goldmann DA Kasper DL Tzianabos AO Pier GB Isolation and chemical characterization of a capsular polysaccharide antigen shared by clinical isolates of Enterococcus faecalis and vancomycin-resistant Enterococcus faecium Infect Immun 1999 67 3 1213 1219 10024563 10.1128/iai.67.3.1213-1219.1999 PMC96449

[B34] Callegan MC Jett BD Hancock LE Gilmore MS Role of hemolysin BL in the pathogenesis of extraintestinal Bacillus cereus infection assessed in an endophthalmitis model Infect Immun 1999 67 7 3357 3366 10377113 10.1128/iai.67.7.3357-3366.1999 PMC116518

[B35] Arnaud M Chastanet A Debarbouille M New vector for efficient allelic replacement in naturally nontransformable, low-GC-content, gram-positive bacteria Appl Environ Microbiol 2004 70 11 6887 6891 10.1128/AEM.70.11.6887-6891.2004 15528558 PMC525206

