# Supplementary material for: Deletion of the glycosyltransferase bgsB of Enterococcus faecalis leads to a complete loss of glycolipids from the cell membrane and to impaired biofilm formation
Source: BMC Microbiol. 2011 Apr 6;11:67. doi: 10.1186/1471-2180-11-67 (PMC3083329; doi:10.1186/1471-2180-11-67)
Supplement: Additional file 1 — Transmission electron microscopy of E. faecalis strains. E. faecalis 12030 wild type (A) and 12030ΔbgsB (B). Bar represents 500 nm. [file 1471-2180-11-67-S1.PDF]

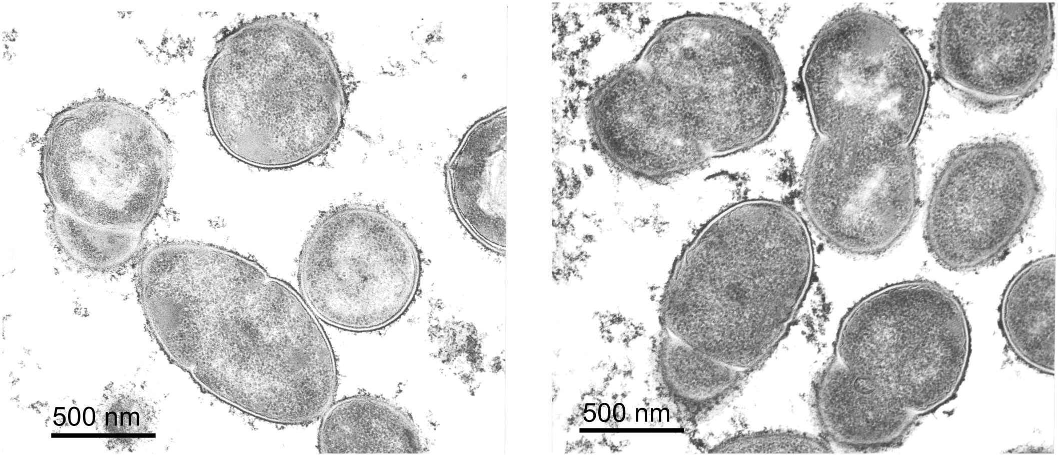

Additional file 1: Transmission electron microscopy of *E. faecalis* strains. *E. faecalis* 12030 wild type (A) and 12030Δ*bgsB* (B). Bar represents 500 nm.
