# Supplementary material for: Deletion of the glycosyltransferase bgsB of Enterococcus faecalis leads to a complete loss of glycolipids from the cell membrane and to impaired biofilm formation
Source: BMC Microbiol. 2011 Apr 6;11:67. doi: 10.1186/1471-2180-11-67 (PMC3083329; doi:10.1186/1471-2180-11-67)
Supplement: Additional file 2 — Autolysis and opsonization of E. faecalis 12030ΔbgsB. A Spontaneous bacterial autolysis. Cells were grown to mid-log phase, resuspended in 10 mM sodium phosphate buffer containing 5% Triton X-100 and the decrease of the OD 600 at 30°C was recorded over time. B Bacterial killing in vitro after 90 min in the presence of 6.5% rabbit complement (white bar), 2 × 10^7^ human PMN plus complement (gray bar) and rabbit antiserum raised against whole bacterial cells (serum dilution 1:2500) plus PMN and complement (black bar). Bars represent means ± SEM. [file 1471-2180-11-67-S2.PDF]

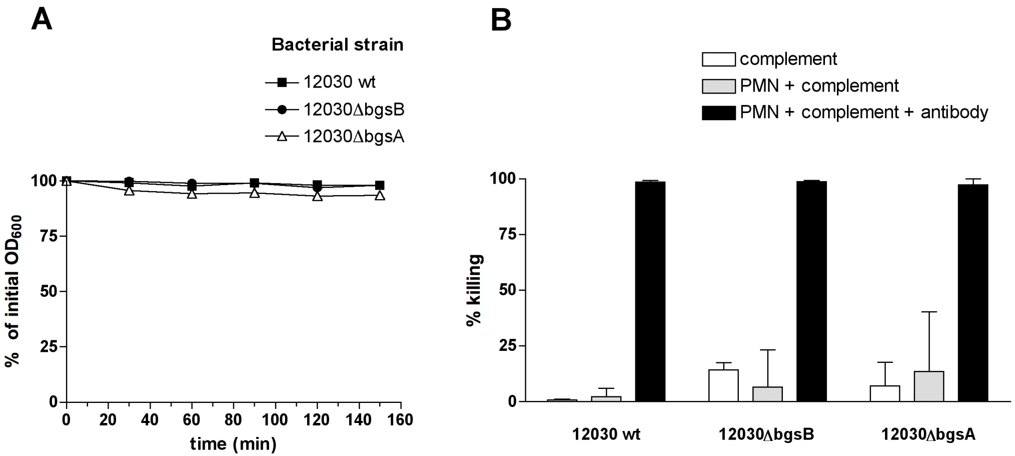

Additional file 2: Autolysis and opsonization of *E. faecalis* 12030Δ*bgsB*.
A Spontaneous bacterial autolysis. Cells were grown to mid-log phase, resuspended in 10 mM sodium phosphate buffer containing 5% Triton X-100 and the decrease of the OD 600 at 30ºC was recorded over time. **B** Bacterial killing in vitro after 90 min in the presence of 6.5% rabbit complement (white bar), 2 x 10^7 human PMN plus complement (gray bar) and rabbit antiserum raised against whole bacterial cells (serum dilution 1:2500) plus PMN and complement (black bar). Bars represent means ± SEM.
