# Supplementary material for: Deletion of the glycosyltransferase bgsB of Enterococcus faecalis leads to a complete loss of glycolipids from the cell membrane and to impaired biofilm formation
Source: BMC Microbiol. 2011 Apr 6;11:67. doi: 10.1186/1471-2180-11-67 (PMC3083329; doi:10.1186/1471-2180-11-67)
Supplement: Additional file 3 — Characterization of E. faecalis ΔbgsB cell walls. A Thin-layer chromatography of cell membrane total lipid extracts of E. faecalis 12030 wild type (lane 1 and 4), 12030ΔbgsB (lane 2 and 5), 12030ΔbgsA (lane 3 and 6). TLC plates were developed using a solvent system of CHCl3/MeOH/H20 (65:25:4, v/v/v). Staining lane 1 - 3 molybdenum blue, lane 4 - 6 ninhydrin. B SDS PAGE of bacterial whole protein extracts. The material was extracted by disrupting the cells with glass-beads, boiling in Laemmli buffer, separated by 4-12% Bis-Tris gels and stained with Coomassie blue. [file 1471-2180-11-67-S3.PDF]

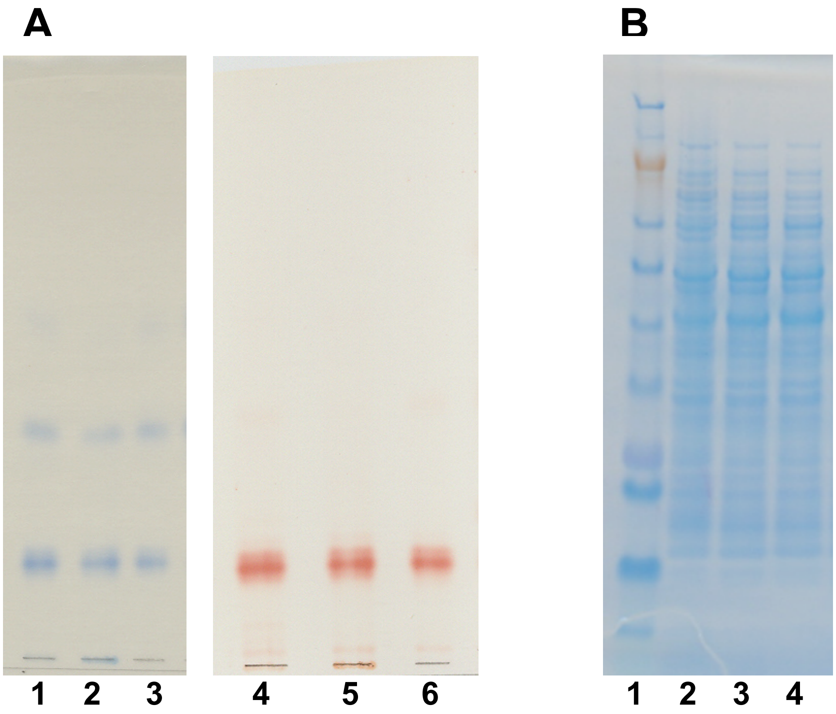

Additional file 3: Characterization of *E. faecalis* Δ*bgsB* cell walls. A Thin-layer chromatography of cell membrane total lipid extracts of *E. faecalis* 12030 wild type (lane 1 and 4), 12030Δ*bgsB* (lane 2 and 5), 12030Δ*bgsA* (lane 3 and 6). TLC plates were developed using a solvent system of $CHCl_3$/MeOH/H_20 (65:25:4, v/v/v). Staining lane 1 - 3 molybdenum blue, lane 4 – 6 ninhydrin. **B** SDS PAGE of bacterial whole protein extracts. The material was extracted by disrupting the cells with glass-beads, boiling in Laemmli buffer, separated by 4-12% Bis-Tris gels and stained with Coomassie blue.
