# Supplementary material for: Deletion of the glycosyltransferase bgsB of Enterococcus faecalis leads to a complete loss of glycolipids from the cell membrane and to impaired biofilm formation
Source: BMC Microbiol. 2011 Apr 6;11:67. doi: 10.1186/1471-2180-11-67 (PMC3083329; doi:10.1186/1471-2180-11-67)
Supplement: Additional file 4 — Minimal bactericial concentration of E. faecalis strains against antimicrobial peptides. Concentrations are expressed as μg/ml. [file 1471-2180-11-67-S4.PDF]

Strain	Nisin	Polymyxin B	Colistin
12030 wild type	64	16	31
12030Δ*bgsB*	64	8	16
12030Δ*bgsA*	64	16	32

Additional file 4: **Minimal bactericial concentration of *E. faecalis* strains against antimicrobial peptides.** Concentrations are expressed as µg / ml.
